# Value of Exome Sequencing in Diagnosis and Management of Recurrent Non-immune Hydrops Fetalis: A Retrospective Analysis

**DOI:** 10.3389/fgene.2021.616392

**Published:** 2021-04-09

**Authors:** Xinyao Zhou, Jia Zhou, Xing Wei, Ruen Yao, Yingjun Yang, Linbei Deng, Gang Zou, Xietong Wang, Yaping Yang, Tao Duan, Jian Wang, Luming Sun

**Affiliations:** ^1^Department of Fetal Medicine, Shanghai First Maternity and Infant Hospital, Tongji University School of Medicine, Shanghai, China; ^2^Department of Medical Genetics and Molecular Diagnostic Laboratory, Shanghai Children’s Medical Center, Shanghai Jiao Tong University School of Medicine, Shanghai, China; ^3^Department of Obstetrics and Gynaecology, Shandong Provincial Hospital Affiliated to Shandong University, Jinan, China; ^4^Department of Molecular and Human Genetics, Baylor College of Medicine, Houston, TX, United States; ^5^AiLife Diagnostics, Pearland, TX, United States

**Keywords:** exome sequencing, single gene disorders, prenatal diagnose, non-immune hydrops fetalis NIHF, prenatal management

## Abstract

The purpose of the study was to use exome sequencing (ES) to study the contribution of single-gene disorders to recurrent non-immune hydrops fetalis (NIHF) and retrospectively evaluate the value of genetic diagnosis on prenatal management and pregnancy outcome. From January 2012 to October 2018, a cohort of 28 fetuses with recurrent NIHF was analyzed by trio ES. Fetuses with immune hydrops, non-genetic factors (including infection, etc.), karyotype, or CNV abnormalities were excluded. Variants were interpreted based on ACMG/AMP guidelines. Fetal therapy was performed on seven fetuses. Of the 28 fetuses, 10 (36%) were found to carry causal genetic variants (pathogenic or likely pathogenic) in eight genes (*GBA*, *GUSB*, *GBE1*, *RAPSN*, *FOXC2*, *PIEZO1*, *LZTR1*, and *FOXP3*). Five (18%) fetuses had variant(s) of uncertain significance (VUS). Of the 10 fetuses with definitive molecular diagnosis, five (50%) were diagnosed with inborn errors of metabolism. Among the seven fetuses who received fetal therapy, two had definitive molecular diagnosis and resulted in neonatal death. Among the remaining five fetuses with negative results, four had newborn survival and one had intrauterine fetal death. Trio ES could facilitate genetic diagnosis of recurrent NIHF and improve the prenatal management and pregnancy outcome.

## Introduction

Hydrops fetalis is a condition of excessively pathological fluid accumulation in more than two fetal tissues and body cavities. It affects 1 in 1,700–3,000 pregnancies and is a life-threatening fetal situation. Non-immune hydrops fetalis (NIHF) was described as fetal hydrops not caused by red cell alloimmunization. NIHF accounts for around 90% of cases of hydrops fetalis (Society for Maternal-Fetal Medicine, Norton et al., 2015).

NIHF should be thought of as a symptom or an end-stage status of a variety of diseases. The etiologies of NIHF include genetic disorders, structural abnormalities, hematologic diseases, infections, twin-twin transfusion syndrome, extrathoracic tumors, and other causes. Genetic disorders, including chromosomal abnormalities, copy number variations (CNV), and single-gene disorders, have been reported to account for one-third of NIHF cases (Moreno et al., 2013; Society for Maternal-Fetal Medicine, Norton et al., 2015).

Traditional karyotyping and chromosomal microarray analysis (CMA) have been suggested as a routine genetic testing offered to NIHF cases according to the AJOG guideline (Santo et al., 2011; Bellini et al., 2015; Society for Maternal-Fetal Medicine, Norton et al., 2015; Mardy et al., 2019, 2020; Sparks et al., 2019). According to recently published study, in which underlying genetic etiology were investigated in a total of 65 NIHF cases, CMA and/or karyotype testing were offered on 67.7% (44/65) of cases, and 25.0% (11/44) were confirmed to have chromosomal abnormalities and pathogenic/likely pathogenic CNV (Sparks et al., 2019). As we have known, a number of single-gene disorders were also associated with NIHF according to previous limited case reports and small series focusing on some specific genetic disorders. For example, lysosomal storage disorders (LSDs) have been reported to contribute to approximately 1% of non-immune hydrops cases by specific enzymatic analyses according to previous studies from 1979 to 2013 (Burin et al., 2004; Bellini et al., 2015). With exome sequencing (ES) widely used in recent years, more rare genetic diseases related to NIHF, such as generalized lymphatic dysplasia, Cornelia de Lange syndrome, Kabuki syndrome, and RASopathies, can also be identified with diagnostic yield of 9% and 29% for NIHF with normal karyotyping and CMA given the results from the PAGE study and the recent publication (Fotiou et al., 2015; Datkhaeva et al., 2018; Lord et al., 2019; Sparks et al., 2020). However, the PAGE study was not designed specific for NIHF cases, and the population studied in the recent publication was of different racial or ethnic backgrounds.

The purpose of the study was to investigate the detection rate and classification of single-gene disorder for recurrent NIHF cases presenting at our Fetal Medicine Unit of Shanghai First Maternity and Infant Hospital for possible fetal therapy by prenatal ES to help prenatal counseling, which were more likely associated with single-gene disorder.

## Materials and Methods

We performed a retrospective study of 49 cases with recurrent fetal hydrops at the Fetal Medicine Unit and Prenatal Diagnosis Center of Shanghai First Maternity and Infant Hospital from January 2012 to October 2018.

Routine prenatal work-up was followed to explore the etiology of fetal hydrops according to AJOG and Chinese NIHF national guideline (Medicine CSOP, 2017; Mardy et al., 2019). ES was offered to the cases prenatally or postnatally as follows: (1) Immune fetal hydrops were ruled out by antibody screening test. (2) Normal karyotyping and CNV results. (3) There was no evidence of intrauterine cytomegalovirus (CMV), toxoplasmosis, and parvovirus infection by serum screening or diagnostic test by polymerase chain reaction (PCR) on amniotic fluid. (4) Thalassemia was ruled out by carrier screening and DNA testing following invasive diagnostic procedures. All the cases had detailed ultrasound and fetal echocardiography. Nuchal translucency or cystic hygroma in the first trimester, fetal structural abnormalities (including skeletal, cardiac, renal, intracranial, genital, face profile, etc.), and growth disorders were recorded.

Pretest counseling for prenatal ES was delivered in an intelligible fashion to the parents by trained genetic professionals. Parents were informed to receive disease-causing variants (pathogenic or likely pathogenic). They were also informed to be aware of the possibility of receiving variants of uncertain significance and choose whether to receive an extended analysis report for the fetus as well as for the parents, which included incidental and secondary findings. Results were reported to the parents when a multidisciplinary team of clinical and laboratory geneticists, obstetricians, and genetic counselors reviewed all the variants in relation to the ultrasound scan findings during pregnancy, or after delivery/termination.

This study was approved by the Ethical Committee of Shanghai First Maternity and Infant Hospital. Written informed consents were obtained from all patients who received genetic tests.

### Exome Sequencing and Variant Evaluation

ES and variant evaluation were performed according to experimental procedures described previously (Hu et al., 2018). Genomic DNA was isolated from all samples by using the QIAamp DNA Blood Midi Kit (Qiagen, Hilden, Germany) according to the manufacturer’s protocol. Targeted regions were captured by the SureSelect^*XT*^ Human All Exon V6 (Cat. No. 5190–8864, Agilent Technologies, Santa Clara, CA) kit. NGS was performed on the HiSeq X Ten platform (Illumina, San Diego, CA) according to the manufacturer’s protocol. Paired-end reads were aligned to the GRCh37/hg19 human reference sequence. BAM and VCF files were generated by NextGENe software (SoftGenetics, State College, PA).

Variants were annotated and filtered by Ingenuity Variant Analysis^1^. Common variants were filtered based on their frequencies in the databases of the Genome Aggregation Database (gnomAD)^2^, the Exome Sequencing Project^3^, the 1000 Genomes Project^4^, and an internal database. Forty genes associated with HPO, termed “Non-immune hydrops fetalis HP:0001790,” were first analyzed. If no candidate variant was found, we further analyzed all genes for putative disease-causing variants. Genes associated with inborn errors of metabolism, generalized lymphatic dysplasia, skeletal dysplasia, neurodevelopment disorders, cardiomyopathies, congenital nephritis, mitochondrial mutations, RASopathies, etc., were carefully analyzed. Rare phenotype-related variants were classified according to the American College of Medical Genetics and Genomics/Association for Molecular Pathology (ACMG/AMP) guidelines (Richards et al., 2015). All putative disease-causing variants detected by ES were confirmed by Sanger sequencing. Family members were also examined by PCR and Sanger sequencing to test the origin of the variants. A multidisciplinary team of clinical and laboratory geneticists, obstetricians, and genetic counselors reviewed all the variants in relation to the ultrasound scan findings to make a final decision about the seven variant classifications (e.g., positive-definitive, positive-probable, positive-possible, uncertain-VUS, uncertain-Autosomal Recessive, Single heterozygous variant, uncertain-contributory, and uncertain-other) according to the classification scheme of case-level results by Vora et al. (2017).

## Results

**FIGURE 1:**
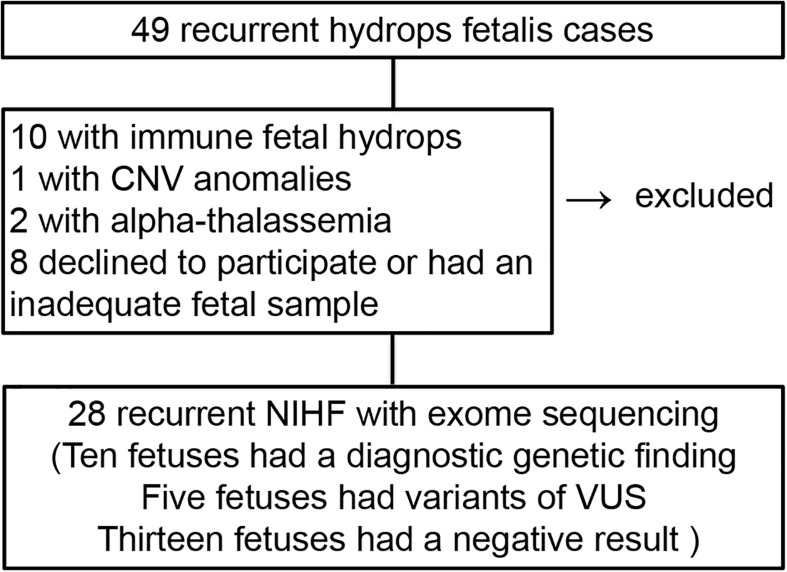
The flow diagram of the cohort and outcomes.

A total of 49 recurrent hydrops fetalis cases were identified from January 2012 through October 2018 in our center (Figure 1). Among these, 10 were immune fetal hydrops cases. One case was CNV anomalies with 5p15.33p14.3 duplication and 13q32.2q34 deletion from paternal balanced translocation. Two cases were confirmed with alpha-thalassemia by carrier screening and DNA testing by invasive diagnostic procedures. There were 28 pregnant women who agreed to have further ES evaluation for hydropic fetuses, and eight women refused. The demographics of the overall cohort are described in Table 1. The turnaround time of ES is 4–6 weeks.

**TABLE 1:** Demographics of the overall cohort.

**Demographics**	**Value**^†^
**Pregnancy history**	
One prior pregnancy affected by NIHF	18/28 (64.3%)
Two prior pregnancies affected by NIHF	9/28 (32.1%)
Three prior pregnancies affected by NIHF	1/28 (3.6%)
**Current pregnancy with NIHF**	
Maternal age (years)	30 (24–41)
Gestational age at hydrops diagnosis (weeks)	23 (14–30.6)
Gestational age at delivery (weeks)^‡^	34.8 (32.9–37.6)
**Other fetal anomaly**	
Cardiovascular	3/28 (10.7%)
Urinary tract	3/28 (10.7%)
Gastrointestinal	3/28 (10.7%)
Skeletal	8/28 (25%)
Polyhydramnios	4/28 (14.3%)
Fetal hydrops plus one other finding	12/28 (42.9%)
Fetal hydrops plus two other finding	4/28 (14.3%)
Fetal hydrops plus two other finding	1/28 (3.6%)
**Prenatal therapeutic management**	
Intrauterine transfusion	3/28 (10.7%)
Needle drainage of effusion	3/28 (10.7%)
Thoracoamniotic shunt placement	3/28 (10.7%)
**Pregnancy outcome**	
Newborn survival	7/28 (25%)
Neonatal death	4/28 (14.3%)
Intrauterine fetal death	1/28 (3.6%)
Termination of pregnancy	16/28 (57.1%)

*^†^Median values (with ranges) shown for continuous variables, percent (N) shown for proportions.*

*^‡^Excluding terminations, spontaneous losses, and stillbirths.*

*NIHF, non-immune hydrops fetalis.*

### Molecular Diagnosis

The genotype and phenotype data of our cohort are listed in Table 2 and Supplementary Table 1. Ten (36%) fetuses had a diagnostic genetic finding (i.e., the variant was considered pathogenic or likely pathogenic and causative of the fetal phenotype). These diagnostic variants included eight missense mutations, two in-frame deletion mutations, two frameshift mutations, one canonical + one splice site mutation, one stop-gain mutation, and one initiation codon mutation. Five (18%) fetuses showed genetic variants that were classified as clinically relevant variants of uncertain clinical significance (VUS). Thirteen fetuses (46%) had a negative result.

**TABLE 2:** The phenotype and genotype information of the cohort.

								**Clinical exome sequencing results**			
**ID**	**Number of prior pregnancies affected by NIHF**	**MA (years)**	**GA (weeks)**	**Fetal hydrops**	**Other ultrasonography abnormalities**	**Prenatal therapeutic management (Y/N)^†^**	**Pregnancy outcome^‡^**	**Variant, Classification and Inheritance^§^**	**Novel or previously reported (PMID, ClinVar)**	**Clinical diagnosis**	**Case-level Classification*^¶^***
1	2	30	20 + 1	Skin edema, pleural effusions, ascites	NT = 1.2 mm, Atrial premature beats, Small stomach bubble	N	TOP at 26 w	*GBA*, *NM_001005741.2*, c.1448T > C, p.Leu483Pro, Pathogenic (PS3 + PM1 + PM2 + PP3 + PP4 + PP5), Homozygous, Paternally and maternally inherited	*PMID: 23719189, 22713811, 15146461*	Gaucher disease, type II, AR	Positive–definitive
2	1	27	24 + 6	Skin edema, pleural effusions, ascites	NT = 1.9 mm, Talipes equinovarus	N	TOP at 27 + 2 w	*GUSB*, *NM_000181.3*, c.1192C > T, Arg398Cys, Likely pathogenic (PM1 + PM2 + PP3 + PP4), Homozygous, Paternally and maternally inherited	*Novel*	Mucopolysaccharidosis VII, AR	Positive–probable
3	2	38	24 + 4	Skin edema, pleural effusions, ascites	NT normal	N	TOP	*GUSB, NM_000181.3*, c.104C > A, p.Ser35*, Pathogenic (PM1 + PM2 + PP3 + PP4), paternally inherited; c.1091C > T, p.Pro364Leu, Likely pathogenic (PM1 + PM2 + PP3 + PP4), Maternally inherited	*ClinVar; PMID:19224584*	Mucopolysaccharidosis VII, AR	Positive–probable
4	2	29	21 + 4	Skin thickening at level of fetal skull, pleural effusions, ascites	NT = 1.7 mm, Talipes equinovarus	Y (Needle drainage of pleural effusion at 23.6 w, Right-side thoracoamniotic shunt placement at 25.5 w, Left-side thoracoamniotic shunt placement 26.5)	Premature rupture of the membranes and cesarean delivery at 31 + 6 w, neonatal mortality within 24 h of birth	*GUSB*, *NM_000181.3*, c.1610T > C, p.Ile537Thr, Likely pathogenic (PM1 + PM2 + PP3 + PP4), paternally inherited; c.323C > T, p.Pro108Leu, Likely pathogenic (PM1 + PM2 + PP3 + PP4), Maternally inherited	*Novel; Novel*	Mucopolysaccharidosis VII, AR	Positive–probable
5	1	30	15 + 3	Skin edema, pleural effusions, ascites	NT = 6.9 mm, Talipes equinovarus	N	TOP	*GBE1*, *NM_000158.3*, c.1229T > G, p.Ile410Arg, Likely pathogenic (PM1 + PM2 + PP3 + PP4), Paternally inherited; c.773C > T, p.Ala258Val, Likely pathogenic (PM1 + PM2 + PP3 + PP4), Maternally inherited	*Novel; Novel*	Glycogen storage disease IV, AR	Positive–probable
6	2	23	26 + 1	Skin edema, pleural effusions	NT = 2.4 mm, Narrow thorax; hand-clenching	N	TOP	*RAPSN*, *NM_005055.4*, c.1119_1121del, p.Lys373del, Likely pathogenic (PM2 + PM3 + PP3 + PP4), Homozygous, Paternally and maternally inherited	*Novel*	Congenital myasthenic syndrome, AR	Positive–probable
7	1	26	25 + 3	Skin edema, pleural effusions	NT normal, Polyhydramnios	Y (Needle drainage of pleural effusion at 25 w, Thoracoamniotic shunt placement at 25 + 3 w, Thoracoamniotic shunt placement at 26.5 w,Intrauterine transfusion at 27 + 4 w)	Cesarean delivery at 32 + 6 w, neonatal mortality within 24 h of birth	*PIEZO1*, *NM_001142864.3*, c.5366_5367dupAG,p. Leu1790Serfs*132, Pathogenic (PVS1 + PM2 + PP4), Paternally inherited; c.7049 + 1G > C, Pathogenic (PVS1 + PM2 + PP3), Maternally inherited	*Novel; Novel*	Lymphatic malformation-6, AR	Positive–definitive
8	2	31	29 + 2	Skin edema, pleural effusions, pericardial effusion, ascites	NT = 1.8 mm	N	TOP	*FOXC2*, *NM_005251.2*, c.361C > T,p.Arg121Cys, Pathogenic (PS2 + PS3 + PM2 + PM5 + PP3 + PP4), *de novo*	*PMID: 19760751*	Lymphedema-distichiasis syndrome, AD	Positive–definitive
9	2	29	14	Skin thickening at level of fetal nucha, pleural effusions	NT = 3 mm, Coarctation of the aorta, Ectopic kidney	N	TOP	*LZTR1*, *NM_006767.3*, c.1A > G, p.Met1?, Pathogenic (PVS1 + PM2 + PP3 + PP4), Paternally inherited; c.27dupG,p.Gln10Alafs*24, Pathogenic (PVS1 + PM2 + PP3), Maternally inherited	*Novel; ClinVar*	Noonan syndrom, AR	Positive–definitive
10	2	27	16 + 5	Skin edema	NT = 1.3 mm	N	TOP	*FOXP3*, *NM_014009.3*, c.1120_1122del, p.Phe374del, Likely pathogenic (PM2 + PM4 + PP3 + PP4), hemizygous, Maternally inherited	*Novel*	Immunodysregulation, polyendocrinopathy, and enteropathy, X-linked	Positive–probable
11	1	27		Skin edema, pleural effusions	NT = 3.1 mm	N	TOP	*RAPSN*, *NM_005055.4*, c.149_153delins24, Pathogenic (PVS1 + PM2 + PP3), Paternally inherited; c.368G > A, p.Gly123Asp, VUS (PM2 + PP3 + PP4), Maternally inherited	*ClinVar; Novel*	Congenital myasthenic syndrome, AR	Positive–possible
12	1	25	21 + 2	Skin thickening at level of fetal skull, ascites	NT = 1.3 mm, Narrow thorax; hand-clenching	N	TOP at 24 + 2 w	*RAPSN*, *NM_005055.4*, c.1119_1121del, p.Lys373del, Likely pathogenic (PM2 + PM3 + PP3 + PP4), Paternally inherited; c.188T > C, p.Leu63Pro, VUS (PM23 + PP3 + PP4), Maternally inherited	*Novel; Novel*	Congenital myasthenic syndrome, AR	Positive–possible
13	1	30	31	Skin thickening at level of fetal occiput and nucha, pleural effusions, pericardial effusion	NT = 1.6 mm	N	Cesarean delivery at 34 w	*PIEZO1*, *NM_001142864.3*, c.4640G > A, p.Arg1547His, VUS (PM2), Paternally inherited; c.6680C > T,p.Ala2227Val, VUS (PM2), Maternally inherited	*Novel; Novel*	Generalized lymphatic dysplasia, AR	Uncertain–VUS
14	1	26	27 + 6	Pleural effusions, ascites	NT = 1.8 mm	N	TOP	*PIEZO1*, *NM_001142864.3*, c.1796T > G, p.Val599Gly, VUS (PM2), Maternally inherited	*Novel*	Dehydrated hereditary stomatocytosis with or without pseudohyperkalemia and/or perinatal edema, AD	Uncertain–VUS
15	3	31	13 + 5	Skin edema, ascites	Cystic hygroma	N	TOP	*PROC*, *NM_000312.3*, c.577_579del p.Lys193del, VUS (PS2 + PM2), *De novo*	*Novel*	Protein C deficiency, AD	Uncertain–VUS

*^†^Y, prenatal therapeutic management was performed on fetuses; N, prenatal therapeutic management was not performed. ^‡^Pregnancy outcome is included TOP, IUFD (Intra uterine fetal death), newborn survival, neonatal death.^§^Classification according to ref. 12 in the text. ^¶^Classification (e.g., positive-definitive, positive-probable, positive-possible, uncertain-VUS, uncertain-AR Het, uncertain-contributory and uncertain-other) was according to the classification scheme of case-level results developed by Vora et al. (2017). MA, maternal age; GA, gestational age; TOP, termination of pregnancy; VUS, variants of uncertain significance.* The italic terms in the column: Variant, Classification, and Inheritance^§^ mean gene name and the corresponding mRNA transcript. The italic terms in the column: Novel or previously reported (PMID, ClinVar) mean that the variant is novel or previously reported in the paper [PubMed identifier (PMID)] and ClinVar database (www.ncbi.nlm.nih.gov/clinvar). The symbol * in GUSB, NM_000181.3,c.104C > A, p.Ser35* means termination codon. The symbol * in PIEZO1, NM_001142864.3, c.5366_5367dupAG, p.Leu1790Serfs*132 means frameshift variant that introduces a termination codon, 132 codons downstream.

Of 10 diagnostic cases, nine fetuses had inherited the relevant mutations from their parents (one fetus with diagnosis of Gaucher disease, type II, *GBA*; three with mucopolysaccharidosis VII, *GUSB*; one with glycogen storage disease IV, *GBE1*; one with congenital myasthenic syndrome, *RAPSN*; one with lymphatic malformation-6, *PIEZO1*; one with Noonan syndrome *LZTR1*, and one with immunodysregulation, polyendocrinopathy, and enteropathy, *FOXP3*). The one with *FOXP3* hemizygous mutation was X-linked recessive inherited disorders. One had a *de novo* mutation (lymphedema–distichiasis syndrome, *FOXC2*). For the *de novo* case given the history of recurrent NIHF, although there was no sample available for the previous pregnancy to confirm the same etiology with current pregnancy, germline mosaicism was highly suspected from parents. Among the 28 fetuses, 15 fetuses had additional abnormal ultrasound findings besides hydrops (including abnormal cardiovascular findings by echocardiogram, urinary tract abnormalities, gastrointestinal anomalies, skeletal abnormalities, and with 14% of the cases (4 of 28) had increased nuchal translucency (3 mm) or cystic hygroma). Among these, 6 (40%) of 15 cases had diagnostic genetic finding from ES results. Thirteen fetuses were isolated NIHF and 4 (31%) of 13 had diagnostic genetic finding. There was no significant difference in the detection rate of single-gene disorders between hydropic fetuses with or without other abnormal ultrasound findings.

### Pregnancy Outcome and the Value of Genetic Diagnosis on Prenatal Management

Among the cohort, 16 chose termination of pregnancy (TOP) directly and 12 families chose to continue pregnancy. Seven fetuses received therapy, including intrauterine transfusion(s) for anemia and centesis and/or shunt insertion for pleural effusion (Tables 1, 2). With ES performed prenatally and postnatally. Five fetal interventions were done in isolated cases. Two were associated with other abnormal ultrasound findings, which were considered as secondary findings related to fetal hydrops: One was associated with talipes equinovarus and one with tricuspid regurgitation. Four of them survived with good outcomes and negative ES results. Three of them had perinatal death. One had fetal demise mostly like due to the complication from blood transfusion with negative ES result. The other two had neonatal death with positive ES results.

## Discussion

Trio-ES could deliver a diagnostic yield of 36% (10/28) in recurrent NIHF fetuses with no aneuploidy and causative CNVs detected and negative screening for alpha-thalassemia and beta-thalassemia.

A higher diagnostic rate (36%) was noticed in our study compared with other studies (Moreno et al., 2013; Sheth et al., 2017; Sudrie-Arnaud et al., 2018; Lord et al., 2019; Sparks et al., 2019, 2020). One reason was that recurrent fetal hydrops in the study represented a highly selected study group, which indicated that a higher incidence of single-gene disorders and non-genetic reasons contributing to NIHF has been ruled out as much as possible in the study following systematic work-up for NIHF. To our knowledge, only one study showed higher diagnostic rate (58%) by prenatal ES than our study. However, the study only included 12 NIHF cases, and 5 of 12 were found to carry the same homozygous variant in the AARS2 gene based on a founder effect (Bruwer et al., 2018). In our study, thalassemia, which can be contributed to NIHF with a higher prevalence in Southern China was ruled out through screening and targeted gene panel. Our data showed two cases (2/39, 5%) with alpha-thalassemia. The diagnostic rate of single-gene disorder by ES for recurrent NIHF in our study would be higher if thalassemia was also taken into account. Nearly half of the cases in our study still remain underdiagnosed. Most the known phenotypes are from postnatal data, making phenotype correlation for prenatal cases difficult. Systematic prenatal and postnatal examination should be evaluated for these NIHF cases. Moreover, undetected variants in critical functional regions of the known genes associated with NIHF beyond the exons or those that were not captured at sufficient depth for panel sequencing and CNVs below the resolution of chromosomal microarray still cannot be excluded. Additional WGS or Bionano optical mapping (BOM) may be beneficial in this situation while considering recurrent NIHF.

Among the cases with positive ES results, 80% of the cases (8 of 10) were autosomal recessive. One was X-linked recessive. One was autosomal dominant. Those major causes of fetal hydrops such as RASopathies, which composed the largest proportion in the recent publication (Sparks et al., 2020), are likely to be *de novo* and therefore would not have been identified in our recurrent cohort, which might skew results toward the autosome recessive inherited disorders. The results also indicated that inborn errors of metabolism accounted for 50% of the single-gene disorders for NIHF detected by ES, and they all confirmed to diagnosed with lysosomal storage disorders (LSD). A previous study from the Indian study group also indicated that lysosomal storage disorders (LSD) accounted for 21% (7/33) of NIHF cases. These indicated biochemical screening (HPLC-MS/MS) and/or lysosomal enzyme testing as a first-line test, which may help to improve the diagnostic strategy for NIHF.

It has been a big challenge to counsel with patients with recurrent fetal hydrops prenatally regarding whether genetic testing, including karyotype, CMA, and ES, should be offered to all the patients with NIHF or not. The study demonstrated that there was no significant difference in the detection rate of single-gene disorders between hydropic fetuses with or without structural abnormalities and suggested that ES should be offered to NIHF, especially recurrent NIHF cases, no matter whether the hydrops was isolated or not.

To explore the value of WES in the fetal therapy, prenatal and postnatal WES results from seven cases following fetal therapy for NIHF were reviewed, and perinatal outcomes were followed up. Four cases survived with good outcomes, which all had negative ES results. Three cases had perinatal death, two of which had positive ES results diagnosed by postnatal samples. Our study indicated that developing rapid or even express ES would be helpful to prioritize workflow for prenatal management for improving the perinatal outcomes.

Our study is not without limitations. The turnaround time (4–6 weeks) is relatively long for a prenatal application, and the sample size in our study is too small to systematically evaluate the profile and frequency of each single-gene disorder in NIHF. A large prospective study of the NIHF cohort will be expected in the future study.

In conclusion, trio-ES can deliver a high diagnostic yield in recurrent NIHF condition. Inborn errors of metabolism were the major causes of the single-gene disorders for recurrent NIHF and accounts for half of our diagnosed cases. The identification of a single-gene disorder may optimize the workflow for prenatal management for NIHF.

## Data Availability Statement

The data that support the findings of this study are available on request from the corresponding author.

## Ethics Statement

The studies involving human participants were reviewed and approved by the Ethical Committee of Shanghai First Maternity and Infant Hospital. Written informed consent to participate in this study was provided by the participants’ legal guardian/next of kin. Written informed consent was obtained from the individual(s), and minor(s)’ legal guardian/next of kin, for the publication of any potentially identifiable images or data included in this article.

## Author Contributions

LS conceived and designed the workflow. XZ performed the experiments, analyzed the data, and wrote the manuscript. JZ, XWe, YiY, LD, and GZ collected the samples. RY and JW analyzed the data and created the figures. XWa, YaY, TD, and JW revised the manuscript. All authors approved the final manuscript.

## Conflict of Interest

YaY was employed by the company AiLife Diagnostics. The remaining authors declare that the research was conducted in the absence of any commercial or financial relationship that could be construed as a potential conflict of interest.

## Footnotes

1 https://variants.ingenuity.com

2 http://gnomad-old.broadinstitute.org/

3 https://esp.gs.washington.edu

4 http://www.1000genomes.org
