# Supplementary material for: Value of Exome Sequencing in Diagnosis and Management of Recurrent Non-immune Hydrops Fetalis: A Retrospective Analysis
Source: Front Genet. 2021 Apr 9;12:616392. doi: 10.3389/fgene.2021.616392 (PMC8063045; doi:10.3389/fgene.2021.616392)
Supplement: Supplementary file 1 [file Data_Sheet_1.DOCX]

**Value of exome sequencing in diagnosis and management of recurrent nonimmune hydrops fetalis: A retrospective analysis**

**Running title**: Exome sequencing in recurrent NIHF and its value on management

Xinyao Zhou^1,#^, Jia Zhou^1,#^, Xing Wei^1^, Ruen Yao^2^, Yingjun Yang^1^, Linbei Deng^1^, Gang Zou^1^, Xietong Wang^3^, Yaping Yang^4,5^, Tao Duan^1^, Jian Wang^2,*^, Luming Sun^1,*^

^1^Department of Fetal Medicine, Shanghai First Maternity and Infant Hospital, Tongji University School of Medicine, Shanghai, China;

^2^Department of Medical Genetics and Molecular Diagnostic Laboratory, Shanghai Children's Medical Center, Shanghai Jiao Tong University School of Medicine, Shanghai, China;

^3^Department of Obstetrics and Gynaecology, Shandong Provincial Hospital Affiliated to Shandong University, Jinan, China;

^4^Department of Molecular and Human Genetics, Baylor College of Medicine, Houston, TX, USA;

^5^AiLife Diagnostics, Pearland, TX, USA.

^#^These authors contributed equally to this work.

^*^Corresponding authors:

Luming Sun, Department of Fetal Medicine, Shanghai First Maternity and Infant Hospital, Tongji University School of Medicine, GaoKe west Road No. 2699, Shanghai, 201204, China. TEL:86-021-20261421, E-mail: luming_sun@163.com

Jian Wang, Department of Medical Genetics and Molecular Diagnostic Laboratory, Shanghai Children's Medical Center, Shanghai Jiao Tong University School of Medicine, Dongfang Road No.1678, Shanghai, China. TEL:86-021-20261421, E-mail: [Labwangjian@shsmu.edu.cn](mailto:Labwangjian@shsmu.edu.cn)

| **Supplementary Table 1 The phenotype and genotype information of the cohort with negative results** | | | | | | | | | | |  |
| --- | --- | --- | --- | --- | --- | --- | --- | --- | --- | --- | --- |
| Case | Number of prior pregnancies affected by NIHF | MA (years) | GA (weeks) | Fetal hydrops | Other ultrasonography abnormalities | Prenatal therapeutic management(Y/N) *^†^* | Pregnancy outcome***^‡^*** | | Fetal karyotype,CMA, infections testing and maternal MCV | Clinical exome sequencing results |  |
| 16 | 1 | 25 | 27+1 | Skin edema, pleural effusions, ascites | NT normal,  Polyhydramnios | Y(Intrauterine transfusion at 27+6w) | | IUFD | Negative | Negative | |
| 17 | 1 | 24 | 30+3 | Skin edema, pleural effusions | NT=2mm,  Polyhydramnios | N | | Cesarean delivery at 33+6w, newborn survival | Negative | Negative | |
| 18 | 1 | 33 | 21+5 | Skin edema, pleural effusions | NT=1.8mm, | N | | TOP at 22w | Negative | Negative | |
| 19 | 2 | 30 | 25 | Skin thickening at level of fetal skull, pleural effusions | NT normal,  Small stomach bubble, small kidney, talipes equinovarus | N | | TOP at 25+2w | Negative | Negative | |
| 20 | 1 | 28 | 21+4 | Skin edema, pleural effusions | NT=1.6mm,  Feet abnormality | N | | TOP | Negative | Negative | |
| 21 | 1 | 41 | 27+6 | Skin thickening at level of fetal skull, pleural effusions, pericardial effusion, ascites | NT normal,  Polyhydramnios | Y(Intrauterine transfusion at 28+2w and 30+1w ) | | Cesarean delivery at 32+6w, newborn survival | Negative | Negative | |
| 22 | 2 | 37 | 24+1 | Skin edema, pleural effusions | NT=1.2mm,  Tricuspid regurgitation | Y(Intrauterine transfusion twice ) | | Cesarean delivery at 34+5w, newborn survival | Negative | Negative | |
| 23 | 1 | 24 | 25 | Pleural effusions, pericardial effusion | NT=1.6mm, | Y(Intrauterine transfusion three times) | | Cesarean delivery at 37+3w, newborn survival | Negative | Negative | |
| 24 | 1 | 31 | 17+6 | Skin thickening at level of fetal skull, pleural effusions, ascites | NT normal | Y(Needle drainage of pleural effusion; thoracoamniotic shunt placement; Needle drainage of ascites; amnioreduction) | | Delivery at 37+4w, newborn survival | Negative | Negative | |
| 25 | 1 | 30 | 30+4 | Pleural effusions, ascites | NT=1.1mm,  Absent of stomach bubble | N | | Cesarean delivery at 32+3w, neonatal death | Negative | Negative | |
| 26 | 1 | 30 | 22+1 | Skin edema, pleural effusions, ascites | NT normal,  Left hydronephrosis | N | | Cesarean delivery at 34+3w, neonatal death | Negative | Negative | |
| 27 | 2 | 27 | 20+2 | Skin edema, pleural effusions, ascites | NT=1.1mm | N | | TOP | Negative | Negative | |
| 28 | 1 | 26 | 25 | Skin edema, pleural effusions, ascites | NT=0.9mm Hand-clenching | N | | TOP | Negative | Negative | |

*^†^* Y, prenatal therapeutic management was performed on fetuses; N, prenatal therapeutic management was not performed.

*^‡^* Pregnancy outcome is included TOP, IUFD (Intra uterine fetal death), newborn survival, neonatal death.
